# Mitochondrial Stress Response Gene *Clpp* Is Not Required for Granulosa Cell Function

**DOI:** 10.3390/antiox10010001

**Published:** 2020-12-22

**Authors:** Ecem Esencan, Mauro Cozzolino, Gizem Imamoglu, Emre Seli

**Affiliations:** 1 Department of Obstetrics, Gynecology and Reproductive Sciences, Yale School of Medicine, New Haven, CT 05610, USA; ecem.esencan@yale.edu (E.E.); mauro.cozzolino@yale.edu (M.C.); aysegulgizem.imamoglu@yale.edu (G.I.); 2 Department of Obstetrics and Gynecology, Rey Juan Carlos University, 28933 Madrid, Spain

**Keywords:** mitochondrial stress, mitochondrial UPR, ovarian aging

## Abstract

Mitochondrial unfolded protein response (UPR^mt^) is a highly conserved mechanism, which is activated upon cellular or metabolic stress and aims to help cells maintain homeostasis. CLPP (caseinolytic peptidase P) plays a crucial factor for UPR^mt^; it promotes the degradation of unfolded mitochondrial proteins. Global germline deletion of *Clpp* in mice results in female infertility and accelerated follicular depletion. Here, we asked whether CLPP is necessary for granulosa/cumulus cell function. *Clpp^flox/flox^* mice were generated and crossbred with *Cyp19a1-Cre* mice to generate mice with granulosa/cumulus cell-specific *Clpp* deletion (*Clpp^−/−^*). Mature (8-week-old) *Clpp^−/−^* female mice (8-week-old) were compared to same age wild type (WT) mice. We found that mature *Clpp^−/−^* female mice were fertile and produced a similar number of pups per litter compared to WT. Folliculogenesis was not affected by the loss of CLPP in granulosa/cumulus cells as *Clpp^−/−^* and WT mice had a similar number of primordial, primary, secondary, early antral, and antral follicles. The number of germinal vesicles (GV) and MII oocytes collected from *Clpp^−/−^* and WT female mice were also similar. Our findings demonstrate that fertility in female mice is not affected by granulosa/cumulus cell-specific UPR^mt^ disruption through CLPP deletion.

## 1. Introduction 

Mitochondria play a central role in cellular energy production. Within the mitochondrial matrix, acetyl CoA molecules derived from glycolysis or fatty acid beta-oxidation enter the Krebs (citric acid) cycle, which results in the generation of reduced forms of NADH and FADH2, in addition to ATP [1]. Then, NADH and FADH2 move to the electron transport chain (ETC) localized at the inner mitochondrial membrane, become reduced to NAD+ and FAD, respectively, and result in further ATP generation through oxidative phosphorylation (OXPHOS) [1]. The aerobic (oxidative) mitochondrial mechanisms involved in energy generation are extremely efficient and result in a net gain of 38 ATP molecules per each glucose molecule metabolized, compared to only two that can be generated through anaerobic glycolysis in the cytoplasm. 

Mitochondrial oxidative metabolism may also result in the generation of reactive oxygen species (ROS), such as hydrogen peroxide (H_2_O_2_). When the amount of ROS is in excess of the cell’s antioxidant response capacity, oxidative stress ensues and may lead to DNA damage and cell death. Mitochondria have several protective mechanisms used to maintain mitochondrial hemostasis, including the mitochondrial unfolded protein response (UPR^mt^), which becomes activated when signs of mitochondrial stress, such as accumulation of unfolded or misfolded proteins, are detected [2,3,4,5]. As initially characterized in Caenorhabditis elegans, UPR^mt^ causes increased expression of mitochondrial CLPP protease (Caseinolytic peptidase P), which cleaves misfolded proteins [6,7,8]. Cleavage of misfolded proteins and subsequent transport of cleavage products to the cytosol results in the activation of the transcription factor ATFS1, which then enters the nucleus and induces transcription of mitochondrial chaperones and ROS detoxification enzymes [3,8,9]. In addition, UPR^mt^ induces glycolysis, mitochondrial fission, and coenzyme Q biosynthesis [3,10,11], altering mitochondrial metabolism and dynamics to promote mitochondrial function and cell survival during stress. UPR^mt^ and the role of CLPP are conserved in mammals (reviewed in [12]).

Key metabolic functions of mitochondria make these organelles important determinants of female reproductive function and oocyte and embryo viability [13]. The number, size, and shape of mitochondria appear to be tightly controlled throughout oogenesis and early embryo development [14]. In mice, several models with targeted deletion of mitochondrial function genes result in infertility [15,16,17]. In humans, oocyte ATP production is associated with embryonic development [18], and some studies correlate mitochondrial DNA content of trophoectoderm biopsies obtained from blastocysts in women undergoing in vitro fertilization (IVF) with reproductive potential of euploid embryos [19,20], whereas other studies do not demonstrate such an association [21].

In a recent article, Wang et al. used a mouse model with a global germline deletion of *Clpp* to characterize the role of CLPP in female reproduction. They found that female mice lacking *Clpp* are infertile and their ovaries show accelerated follicular depletion, consistent with diminished ovarian reserve. In addition, these mice generate a lower number of mature oocytes and two-cell embryos, and do not make blastocysts [15]. These findings are consistent with the conclusions of another study that found female mice with global deletion of *Clpp* to be sterile and have altered follicular histology [22]. Although these studies generated highly interesting and relevant data, they did not determine which reproductive cell type (somatic vs. gonadal) is primarily dependent on CLPP for its function. In addition, the role of CLPP in granulosa/cumulus cells under conditions associated with mitochondrial stress has not yet been studied. Therefore, in the current study, we used a targeted deletion strategy that removed *Clpp* in granulosa/cumulus cells.

## 2. Materials and Methods

### 2.1. Animal Breeding and Genotyping

**Figure 1:**
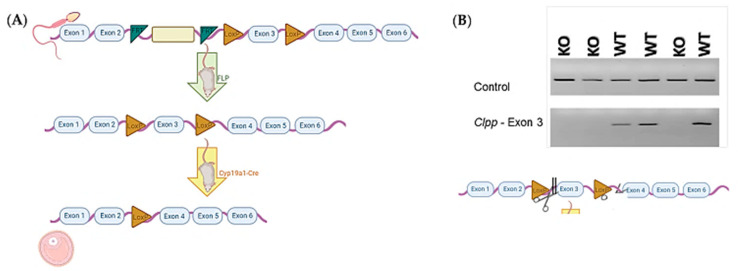
(**A**) Schematic illustration of the generation of mice with targeted deletion of *Clpp* in granulosa cells, using sperm with allele carrying reporter-tagged insertion. (**B**) Example of genotyping results from cumulus cells obtained from *Clpp^−/−^* and WT females (*n* = 3 for each genotype), using polymerase chain reaction (PCR). The upper frame shows control using primers for a constitutively expressed mitochondrial gene *Mfn2*. The lower frame shows the PCR product generated using primers upstream and downstream of exon 3, between the exon and LoxP.

Sperm carrying mutant *Clpp* allele in C57BL/6 background with reporter-tagged insertion were purchased from Wellcome Trust Sanger Institute (Colony ID: EPD0665_3_D05) and used to fertilize wild-type (WT) female mice oocytes through in vitro fertilization (IVF). WT surrogate carriers were used to generate offspring (*n* = 6). Half of the offspring (*n* = 3) carrying the mutant allele were crossbred with a mouse carrying FLP gene following vendor instructions to obtain mice with *Clpp*^flox^ allele. Female mice (*n* = 3) with *Clpp*^flox^ allele were fertilized with sperm with Cyp19a1-Cre (where Cre is driven by Cyp19a1 promoter), which was obtained from the University of California Davis (stock number: 037056-UCD-SPERM) to obtain a generation of mice with *Clpp^flox^/Cyp19a1-Cre* (Figure 1). Colony numbers gradually expanded as needed.

Genotyping was carried out using primers listed in Supplementary Table S1.

For simplicity, mice with cumulus cell-specific Clpp deletion, i.e., *Clpp^flox^/Cyp19a1-Cre* mice, will be referred to as *Clpp^−/−^* hereafter. Female *Clpp^−/−^* mice and their WT litter mates were used for the experiments detailed below. Mice care, breeding, and experimental procedures were conducted according to Yale University animal research requirements, using protocols approved by Institutional Animal Care and Use Committee (protocol #2020-11207).

#### Quantitative Reverse Transcription-Polymerase Chain Reaction (qRT-PCR)

RNA was extracted from pooled cumulus cells(CCs) obtained from ten cumulus oocyte complexes (COCs) per mice, liver, uterus, and kidney (*n* = 5 for each genotype per experiment) using an RNAqueous Micro Kit (Invitrogen, Carlsbad, CA). Reverse transcription was performed following manufacturer’s instructions in two sequential steps using a RETROscript Kit (Invitrogen, Carlsbad, CA). Briefly, to eliminate any secondary structures, extracted RNA and random decamers were incubated at 85 °C for 3 min. This was followed by adding buffer, reverse transcriptase enzyme, RNAse inhibitor, and dNTP mix and incubating at 42 °C for 1 h. cDNA obtained from this reaction was assessed in triplicate using an iCycler (Bio-Rad Laboratories, Hercules, CA). For each 10 μL reaction, 5 μL SYBR Green Supermix (Bio-Rad Laboratories, Hercules, CA), 1 μL of each primer, 2 μL H2O, and 1 μL cDNA were used. Each experiment was repeated at least three times using different animals of the same genotype each time. The 2^(−ΔΔCT)^ (cycle threshold) method was used to assess relative expression levels after normalization to β-actin levels. Primers used in qRT-PCR are listed in Supplementary Table S1.

### 2.2. Fertility Assessment

To evaluate the fertility of *Clpp^−/−^* female mice, 2-month-old *Clpp^−/−^* and WT (*n* = 6, for each group) were mated with adult WT males with proven fertility. In each cage, one female mouse was hosted with one male mouse for 12 weeks. The number of litters and pups were recorded with daily monitoring.

### 2.3. Histomorphometric Analysis of Folliculogenesis in Ovaries

Histomorphometric analysis was performed on ovarian sections stained with hematoxylin and eosin (H&E). Ovaries from 2-, 6-, and 9-month-old *Clpp^−/−^* and WT (both ovaries of 3 mice for each genotype at each age group) were collected and fixed in 4% (*w/v*) paraformaldehyde in Dulbecco’s phosphate-buffered saline (Sigma, St. Louis, MO, USA) at room temperature overnight. They were then dehydrated and embedded in paraffin. Five micrometer (µm) serial sections were obtained and stained with H&E using standard protocols [23]. Every 5th section (a total of 35 sections per ovary) was marked and assessed. Only follicles containing a visible nucleus were counted to determine the number of primordial, primary, secondary, early antral, and antral follicles as previously described [24]. The total number of follicles counted were reported without normalization. Primordial follicles were defined with an oocyte surrounded by a single layer of squamous granulosa cells. Primary follicles were formed by an oocyte surrounded by a single layer of cuboidal granulosa cells. Secondary follicles contained an oocyte surrounded by two or more layers of cuboidal granulosa cells. Early antral follicles and antral follicles possessed an oocyte with four or more layers of surrounding granulosa cells multiple small antral spaces and one large antral space, respectively.

### 2.4. Oocyte and Cumulus Cell Collection

Germinal vesicle (GV) stage oocytes, oocytes at metaphase of second meiotic division (MII), and cumulus cells were obtained by superovulation of mature 2-month-old *Clpp^−/−^* and WT mice as previously described [25]. To obtain GV stage oocytes, mice were euthanized by CO_2_ inhalation 44 h after intraperitoneal injection of 5IU PMSG. Ovaries were removed and punctured in M2 medium (Sigma, St. Louis, MO, USA) with 10 µM milrinone (Sigma, St. Louis, MO, USA) under dissecting microscope (Olympus SZH-ILLK) with 26-1/2-gauge needle to isolate cumulus-oophorus complexes containing GV stage oocytes and cumulus cells. Oocytes were stripped from neighboring cumulus cells with a mouth pipette and collected in individual tubes. To obtain mature, M2 stage oocytes, 5IU of human chorionic gonadotropin (hCG; Sigma, St. Louis, MO, USA) was injected intraperitoneally 48 h after PMSG injection. Unfertilized MII oocytes were collected from mice oviducts 14 h after hCG injection. To collect fertilized embryos, females were mated with WT males immediately after the hCG injection. The following morning, mating was confirmed by the presence of a vaginal plug. Two-cell embryos were collected 44 h after hCG injection from the oviducts in KSOM medium (Millipore, Darmstadt, Germany). Blastocysts were collected 92 h after hCG injection from uterus into M2 medium (Sigma, St. Louis, MO, USA).

### 2.5. Quantification of mtDNA Copy Number in Cumulus Cells and Oocytes

To quantify mtDNA in cumulus cells and oocytes, *Cox3* plasmid fragment was amplified by subcloning into pCR^TM^2.1-TOPO^®^-colony vector (Invitrogen, Carlsbad, CA, USA), as previously described [14]. One-Shot TOP10 Chemically Competent *E. coli* was transformed with plasmid and grown overnight at 37 °C. The recombinant plasmid was then purified using Qiagen plasmid isolation kit (catalogue number 27104) and the inserted mtDNA fragment was confirmed by DNA sequence analysis. The amount of plasmid DNA was quantified using a NanoDrop 2000 spectrophotometer (Thermo Scientific, Waltham, MA, USA). A standard curve with 10^9^ to 10^2^ plasmid molecules was generated by serial 10-fold dilutions. Individual GV stage oocytes and approximately 50 cumulus cells stripped from cumulus-oophorus complexes of 2-month-old *Clpp^−/−^* and WT mice (*n* = 3) were lysed in 10 µl lysis solution at 55 °C for 2 h. Lysis solution was inactivated at 95 °C for 10 min and the mix was used directly for qPCR in triplicate as previously described [15]. Individual oocytes’ and aggregate cumulus cells’ mtDNA copy numbers were extrapolated from the standard curve.

### 2.6. Determination of ROS Levels

Reactive oxygen species (ROS) generation was induced by exposing COCs from 2-month-old *Clpp^−/−^* and WT mice to 20mM H_2_O_2_ dissolved in M2 medium for 5 min at 37 °C. COCs were then washed 3 times with M2 medium and incubated in M2 medium containing 6-carboxy-2′, 7′-dichlorodihydrofluorescein diacetate (carboxy-H2DCFDA) (Life Technologies, Thermofisher Scientific, Carlsbad, CA, USA). Carboxy-H2DCFDA is a nonfluorescent chemical, which has the ability to pass through the plasma membrane and converts to green fluorescent upon oxidation with ROS [26]. H2DCFDA is a fluorescent probe commonly employed and may react with several ROS, including hydrogen peroxide, hydroxyl radicals, and peroxynitrite. The cell-permeant H2DCFDA passively diffuses into cells and is retained in the intracellular level after cleavage by intracellular esterases. Upon oxidation by ROS, the nonfluorescent H2DCFDA is converted to the highly fluorescent 2’,7’ dichlorofluorescein (DCF) [26]. A quantity of 5 µL of H2DCFDA was dissolved in 1 mL of M2 medium, and COCs were incubated for 20 min at 37 °C in the dark. COCs were then washed with H2DCFDA-free media and images were captured with a Leica SP5 spectral scanning confocal microscope. Fluorescence in images was quantified using Image J software (National Institute of Health). In total, 5 COCs per group were analyzed for each experiment; experiments were repeated 3 times.

### 2.7. Statistical Analysis

All statistical analysis was performed using Graph Pad Prism software, version 8.3.0. The Student’s t-test was used to analyze statistical significance between groups. Quantitative analysis was expressed as mean ± SEM and significance was assessed at *p* < 0.05.

## 3. Results

### 3.1. Lack of Clpp in Granulosa/Cumulus Cells did not Affect Fertility Potential in Mice

The fertility of mature *Clpp^−/−^* mice was similar to that of WT females (Table 1). Loss of CLPP in cumulus cells of female mice did not affect fertility potential compared to same-age WT counterparts. After 12 weeks of mating with male WT mice with proven fertility, the total number of litters (15 vs. 15, *n* = 6) and litters per female mouse (2.50 ± 0.5 vs. 2.50 ± 0.5, *p* = 1) was the same. The total number of pups obtained from *Clpp^−/−^* and WT mothers was also similar (122 vs. 120). There was no significant change in pups per litter (8.13 ± 1.28 vs. 8.00 ± 1.70, *p* > 0.05) between *Clpp^−/−^* and WT female mice.

Fertility of female *Clpp^−/−^* and WT mice (8-week-old, *n* = 4 for each genotype) was assessed by mating with WT males of proven fertility (male/female; 1:1) for 12 weeks.

*Clpp^−/−^* mice had a similar litter size (pups per litter) and litters per female compared with WT females.

### 3.2. Folliculogenesis and Oocyte Maturation was not Altered by Granulosa/Cumulus Cell-Specific CLPP Loss

**Figure 2:**
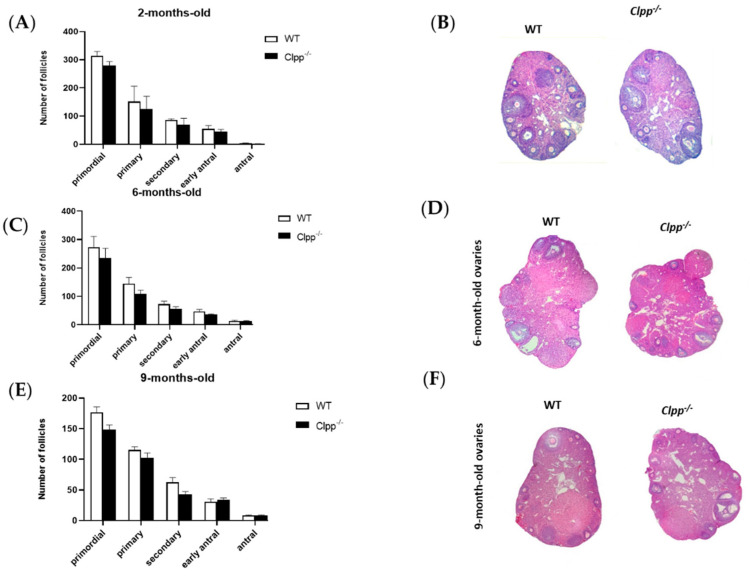
Assessment of follicle development in mice with targeted deletion of Clpp in granulosa cells. (**A**,**C**,**E**) Follicle development was assessed in 2-, 6-, 9-month-old Clpp^−/−^ and WT mice ovaries (both ovaries of three different mice were assessed in each group, and for each timepoint). Data presented as mean ± SEM. (**B**,**D**,**F**) Representative micrographs of 2-, 6-, 9-month-old Clpp^−/−^ and WT mice ovarian sections stained with hematoxylin and eosin.

Folliculogenesis was not affected by loss of CLPP in cumulus cells in young or old mice. In 2-month-old females, the numbers of primordial (278.20 ± 13.08 vs. 317.80 ± 19.90; *p =* 0.69), primary (135.30 ± 31.47 vs. 147.80 ± 88.74; *p =* 0.87), secondary (71.0 ± 26.16 vs. 83.50 ± 3.54; *p =* 0.57), early antral (46.25 ± 13.08 vs. 54.75 ± 12.37; *p =* 0.57), and antral follicles (3.02 ± 2.12 vs. 1.75 vs. 0.35; *p =* 0.5) did not change significantly when the pivotal mtUPR gene was knocked out in cumulus cells (Figure 2A,B). Consistent with these findings, ovarian sizes of 2-month-old *Clpp^−/−^* and WT mice were similar (Supplemental Figure S1).

In 6-month-old mice, the number of primordial (280 ± 39.5 vs. 234.5 ± 24.5; *p =* 0.44), primary (151.5 ± 28.5 vs. 108.5 ± 9.5; *p =* 0.35), secondary (75.5 ± 10.5 vs. 56 ± 6; *p =* 0.29), early antral (47 ± 5 vs. 35 ± 2; *p =* 0.21), and antral follicles (13.5 ± 1.5 vs. 12.5 ± 1.5; *p =* 0.68) did not show any statistical significance between *Clpp^−/−^* and WT (Figure 2C,D).

Similarly, in older mouse, at 9 months of age, the number of primordial (177.5 ± 10.5 vs. 148.5 ± 9.5; *p =* 0.17), primary (116.5 ± 6.5 vs. 101 ± 8; *p =* 0.27), secondary (62.5 ± 5.5 vs. 42.5 ± 3,5; *p =* 0.11), early antral (30.5 ± 3.5 vs. 32 ± 4; *p =* 0.80), and antral follicles (8.5 ± 0.5 vs. 8 ± 1; *p =* 0.71) did not show any statistical significance between *Clpp^−/−^* and WT (Figure 2E,F).

### 3.3. Immature and Mature Oocyte Production was not Altered in Mice with Granulosa/Cumulus Cell-Specific Deletion of Clpp

**Figure 3:**
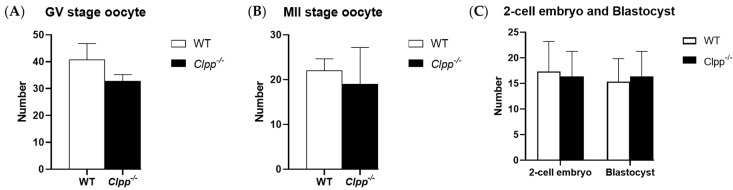
Oogenesis and embryogenesis in Clpp^−/−^ mice. (**A**) Germinal vesicle (GV) stage oocytes were collected from 2-month-old Clpp^−/−^ and WT mice 44–48 h after PMSG injection (five mice were assessed in each group). (**B**) MII stage oocytes were collected from 2-month-old Clpp^−/−^ and WT mice 12–14 h after hCG injection, which was performed 48 h after PMSG injection (five mice were assessed in each group). (**C**) Number of 2-cell embryos and blastocysts in Clpp^−/−^ and WT mice (five mice were assessed in each group). Data presented as mean oocyte or embryo per mouse ± SEM.

GV or MII oocyte production was not affected by CLPP loss in cumulus cells. In 2-month-old mice, *Clpp^−/−^* and WT mice produced a similar number of GV stage oocytes (38.4 ± 8.7 vs. 42.8 ± 5.4; *p =* 0.37). The number of mature, MII stage oocytes was also not significantly different between the two groups (21.3 ± 6.8 vs. 23.3 ± 7.1; *p =* 0.65). Number of 2-cell embryos (16.3 ± 4.9 vs. 17.3 ± 5.8, *p =* 0.83) and blastocysts (16.3 ± 4.9 vs. 15.3 ± 5.5, *p =* 0.810) were not significantly different between *Clpp^−/−^* and WT mice (Figure 3).

### 3.4. Clpp^−/−^ Cumulus Cells did not have any Abnormal mtDNA Copy Number or ROS Production

**Figure 4:**
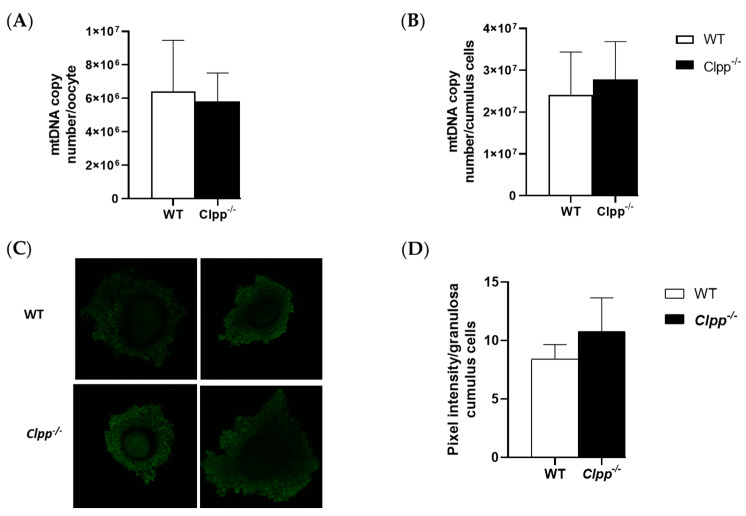
Mitochondrial function in *Clpp^−/−^* oocytes and cumulus cells. (**A**) mtDNA copy number was determined by qPCR in GV stage oocytes collected from 2-month-old *Clpp^−/−^* and WT mice. In each experiment, five mice were included in each group, and five GV oocytes/mice (a total of 25 GV oocytes per experiment) were analyzed. Each experiment was repeated three times. (**B**) mtDNA copy number was determined by qPCR in granulosa/cumulus cells collected from 2-month-old *Clpp^−/−^* and WT mice. In each experiment, five mice were included in each group, and granulosa/cumulus cells obtained from five COCs/mice (a total of 25 COCs per experiment) were analyzed. Each experiment was repeated three times. (**C**) Fluorescence intensity of carboxy-H2DCFDA was used to measure ROS levels in cumulus-oophorus complexes after treatment with H_2_O_2_. (**D**) Pixel intensity in granulosa cumulus cells is reported using Image J. Data presented as mean ± SEM.

Mitochondrial copy number in both cumulus cells and oocytes were similar in mature *Clpp^−/−^* and WT female mice. In cumulus cells, mtDNA copy numbers for *Clpp^−/−^* and WT were 27,793,262 ± 9,070,406 and 24,163,434 ± 10,191,895, respectively (*p =* 0.24). In GV-stage oocytes collected from *Clpp^−/−^* and WT mice, mtDNA numbers were 5,826,568 ± 1,688,408 and 6,422,192 ± 3,047,395, respectively (*p =* 0.45). These parameters were not different between the groups (Figure 4).

Loss of CLPP did not have a significant effect on the ROS production of cumulus cells. The percentage of ROS in cumulus cells of secondary follicles collected from 2-month-old *Clpp^−/−^* and WT mice were not significantly different (10.8 ± 2.86 vs. 8.4 ± 1.24, *p =* 0.13) (Figure 4).

## 4. Discussion

CLPP plays a central role in UPR^mt^ by promoting degradation of unfolded or misfolded mitochondrial proteins. We had previously demonstrated that the global deletion of *Clpp* in mice results in female infertility and accelerated follicular depletion [15]. In the current study, we investigated whether CLPP expression in granulosa/cumulus cells is required for reproductive competence. We found the fertility of mature female mice with targeted deletion of *Clpp* in granulosa/cumulus cells to be similar to that of WT. Our findings suggest that, although CLPP is an essential protein for UPR^mt^ and its global expression is required for female and male fertility [15,22], follicular somatic cell CLPP expression is not a requirement for female fertility.

In the current study, we evaluated follicle development in mice with granulosa/cumulus cell-specific deletion of *Clpp,* throughout their reproductive lifetime. We did not found any impairment in follicle development or the number of primordial, primary, secondary, early antral, or antral follicles at 2, 6, or 9 months. Our findings are in contrast with studies that used global germline deletion of *Clpp*, a strategy that causes the deletion of *Clpp* in all cells [15,22] In one such study, Gispert et al. reported that ovaries of mice with global deletion of *Clpp* were smaller and had reduced layers of follicular granulosa cells with a higher number of apoptotic bodies. They suggested that this finding was consistent with ovarian failure associated with *CLPP* missense and splice-site mutations found in a variant of human Perrault syndrome [27], in which streak ovaries with few scattered follicles had been described [28,29]. In a more recent study, Wang et al. used the same model and confirmed that global *Clpp*-deletion causes female infertility associated with loss of primordial and primary follicles at 6, 9, and 12 months, and increased follicular atresia. Collectively, our findings and the findings in the two prior studies suggest that, although global *Clpp*-deletion causes infertility, granulosa/cumulus cell function is not dependent on CLPP expression. Therefore, the requirement for CLPP must be elsewhere. Although one could speculate that this requirement is in oocytes, this remains to be proven.

Oocytes are unable to metabolize glucose and require pyruvate for survival and maturation [30,31,32,33]. Follicular somatic cells, therefore, play an important role in the nutritional support of the oocyte. Pyruvate is produced from glucose in granulosa/cumulus cells and transported to the oocyte through gap junctions [30,31,32,33]. Not surprisingly, glycolysis is active in cumulus cells, whereas oocytes metabolize pyruvate through oxidative phosphorylation to produce energy for growth and maturation [34]. In addition to pyruvate, the cumulus cells supply the oocyte with amino acids (such as L-alanine) and cholesterol as substrates [35]. The ability of *Clpp*-deficient granulosa/cumulus cells to support reproduction could potentially be explained by the fact that mitochondrial metabolic activity and OXPHOS in granulosa/cumulus cells may not be central to oocyte and follicle viability.

The interactions between the oocyte and neighboring somatic cells that regulate metabolism are bidirectional. The metabolic activity of cumulus cells requires secretion of oocyte-specific paracrine factors (such as fibroblast growth factor 8B (FGF8B), bone morphogenetic protein 15 (BMP15), and growth differentiation factor 9 (GDF9)) [36]. In the current study, we found that the number of immature or mature oocytes produced by female mice with granulosa/cumulus cell-specific targeted deletion of *Clpp* were similar to that of WT. In addition, these oocytes did not have increased ROS or mtDNA, which occur in association with oxidative stress. These findings are in contrast to those found in mice with global deletion of *Clpp*, in which the GV and MII oocytes were decreased, and ROS and mtDNA significantly increased [15,22]. Collectively, these data suggest that the requirement for *Clpp* in reproduction may be in oocytes.

In this study, we built on the work of previous researchers in delineating the role of UPR^mt^ and CLPP in female fertility. Our findings clearly show that female fertility is not disrupted in the absence of CLPP in granulosa/cumulus cells, and suggest that UPR^mt^ and CLPP requirement may be in oocytes. Further studies are needed to delineate the role of UPR^mt^ and CLPP in reproduction and to exploit these pathways for diagnostic and therapeutic purposes.

## 5. Conclusions

Despite the well-described role of granulosa/cumulus cells in oocyte metabolism, our findings demonstrate that *Clpp*-mediated UPR^mt^ in these cells is not required for female fertility. Therefore, female infertility observed in models with global loss of *Clpp* is likely caused by oocyte-related effects, and UPR^mt^ is not a crucial pathway in granulosa/cumulus cells.

## Figures and Tables

**Table 1:** Fertility assessment in female mice with targeted deletion of *Clpp* in granulosa cells.

Genotype	*n*	Litters	Pups	Pups Per Litters	Litters Per Female
WT	6	15	120	8.00 ± 1.70	2.50 ± 0.5
KO	6	15	122	8.13 ± 1.28	2.50 ± 0.5

## Supplementary Materials

The following are available online at https://www.mdpi.com/2076-3921/10/1/1/s1, Supplemental Figure S1. Clpp mRNA expression in somatic tissues and granulosa/cumulus cells of wild type mice (*n* = 5 per tissue), Supplemental Figure S2. Ovarian size in Clpp^−/−^ mice (*n* = 3 different mice assessed in each group). (A) Representative photographs of ovaries from 8-week old Clpp^−/−^ and WT mice. Lines on the ruler are 1 mm apart. (B) Ovarian size displayed as area (length x width), comparing Clpp^−/−^ and WT mice (n = 3 for each genotype). There was no statistically significant difference between the groups, Supplemental Table S1. Primers used for genotyping and RT-PCR.
