# Supplementary material for: Mitochondrial Stress Response Gene Clpp Is Not Required for Granulosa Cell Function
Source: Antioxidants (Basel). 2020 Dec 22;10(1):1. doi: 10.3390/antiox10010001 (PMC7821922; doi:10.3390/antiox10010001)
Supplement: Supplementary file 1 [file antioxidants-10-00001-s001.pdf]

Supplementary materials

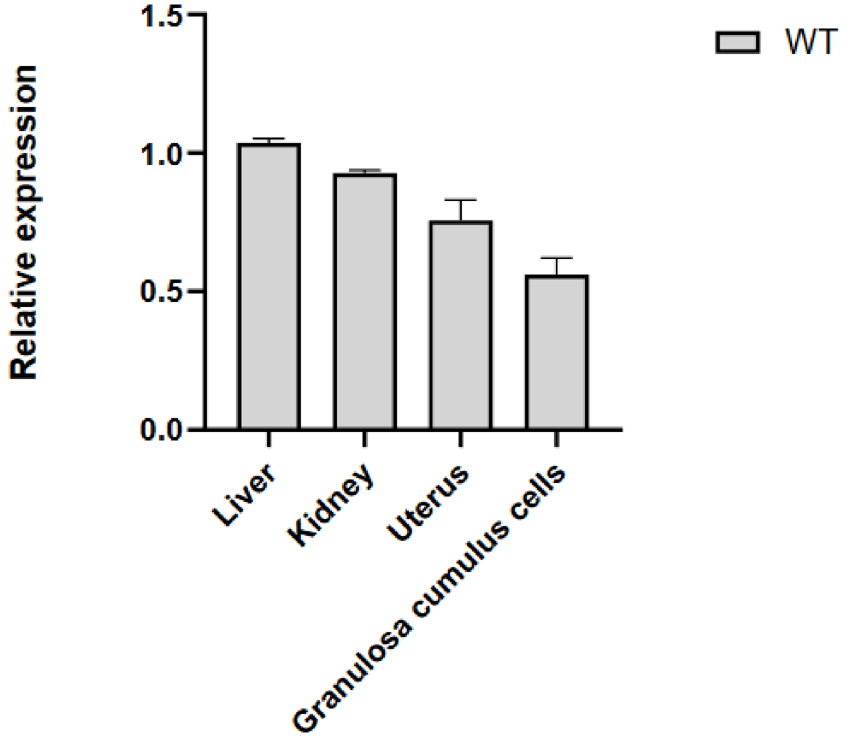

Figure 1. Clpp mRNA expression in somatic tissues and granulosa/cumulus cells of wild type mice (*n* = 5 per tissue)

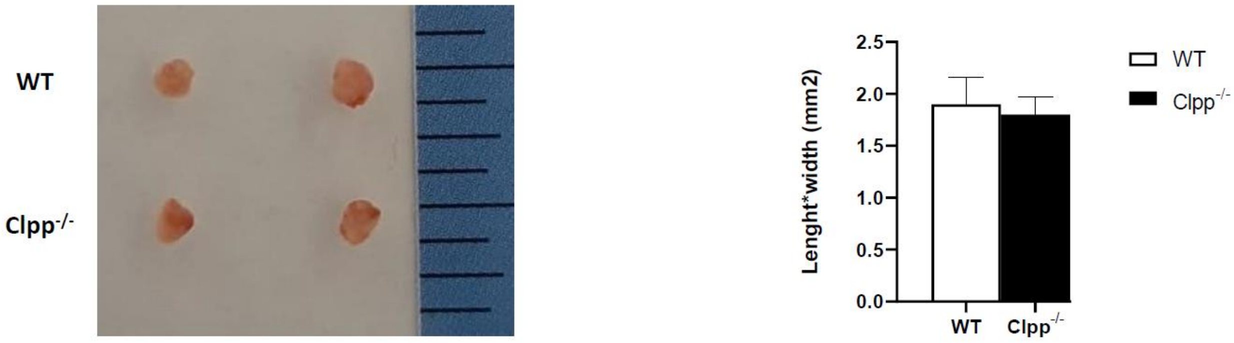

Figure 2. Ovarian size in Clpp−/− mice (*n* = 3 different mice assessed in each group). (**A**) Representative photographs of ovaries from 8-week-old Clpp−/− and WT mice. Lines on the ruler are 1 mm apart. (**B**) Ovarian size displayed as area (length x width), comparing Clpp−/− and WT mice (*n* = 3 for each genotype). There was no statistically significant difference between the groups

Table 1. Primers used for genotyping and RT-PCR

Gene	Primer sequences<br>(5' to 3'; F, forward; R, reverse)
Clpp	F: GAGGCCCTGGGAACCAGGAA<br>R: TCTGCTGTTGTCAGCCATTC
Mfn2	F: GAAGTAGGCAGTCTCCATCG<br>R: AACATCGCTCAGCCTGAACC
Flp	F: CCTAAGGTCCTGGTTCGTCA<br>R: TTGTTGCTTTTTGCGTCTTG
Cox3	F: TTTGCAGGATTCTTCTGAGC<br>R: TGAGCTCATGTAATTGAAACACC
Cre	F: TACAGCACCCTCTGAAGCAA<br>R: ACTTGGTCAAGTCAGTGCG

Abbreviations: *Clpp*: Caseinolytic Peptidase P; *Mfn2*: Mitofusin 2; *Flp*: Flippase; *Cox3*: Cytochrome C Oxidase Subunit III; *Cre*: Cre Recombinase.
